# Does vitamin D protect or treat Parkinson’s disease? A narrative review

**DOI:** 10.1007/s00210-023-02656-6

**Published:** 2023-08-09

**Authors:** Hayder M. Al-kuraishy, Ali I. Al-Gareeb, Hend Mostafa Selim, Athanasios Alexiou, Marios Papadakis, Walaa A. Negm, Gaber El-Saber Batiha

**Affiliations:** 1 Department of Clinical Pharmacology and Medicine, College of Medicine, Mustansiriyah University, Baghdad, 14132 Iraq; 2 Biochemistry Department, Faculty of Pharmacy, Tanta University, Tanta, 31527 Egypt; 3 Department of Science and Engineering, Novel Global Community Educational Foundation, Hebersham, NSW 2770 Australia; 4 AFNP Med, 1030 Wien, Austria; 5 Department of Surgery II, University Hospital Witten-Herdecke, Heusnerstrasse 40, University of Witten-Herdecke, 42283 Wuppertal, Germany; 6 Department of Pharmacognosy, Faculty of Pharmacy, Tanta University, Tanta, 31527 Egypt; 7 Department of Pharmacology and Therapeutics, Faculty of Veterinary Medicine, Damanhour University, Damanhour, 22511 AlBeheira Egypt

**Keywords:** Parkinson’s disease, Vitamin D, Dopaminergic neurons, Oxidative stress

## Abstract

Parkinson’s disease (PD) is a neurodegenerative brain disease (NBD) developed due to dopaminergic neuron loss in the substantia nigra (SN). Vitamin D (VD), VD receptor (VDR), and VD metabolites are highly expressed in the human brain and play a critical role in maintaining different brain functions. VDRs are highly expressed in the SN that regulates the activity of dopaminergic neurons and synaptic plasticity. VD exerts protective and therapeutic effects against the development of PD by modulating dopaminergic neurons of SN. VD reduces oxidative stress and neuroinflammation in PD because of its anti-inflammatory and antioxidant activities. Different studies revealed the protective effect of VD in the management of PD. However, the potential therapeutic effect of VD in well-established PD remains controversial. Therefore, this review aims to elucidate VD’s preventive and therapeutic roles in PD. In conclusion, VD deficiency is associated with increased PD risk, but VD supplementation in well-established PD plays little role.

## Introduction

**Fig. 1:**
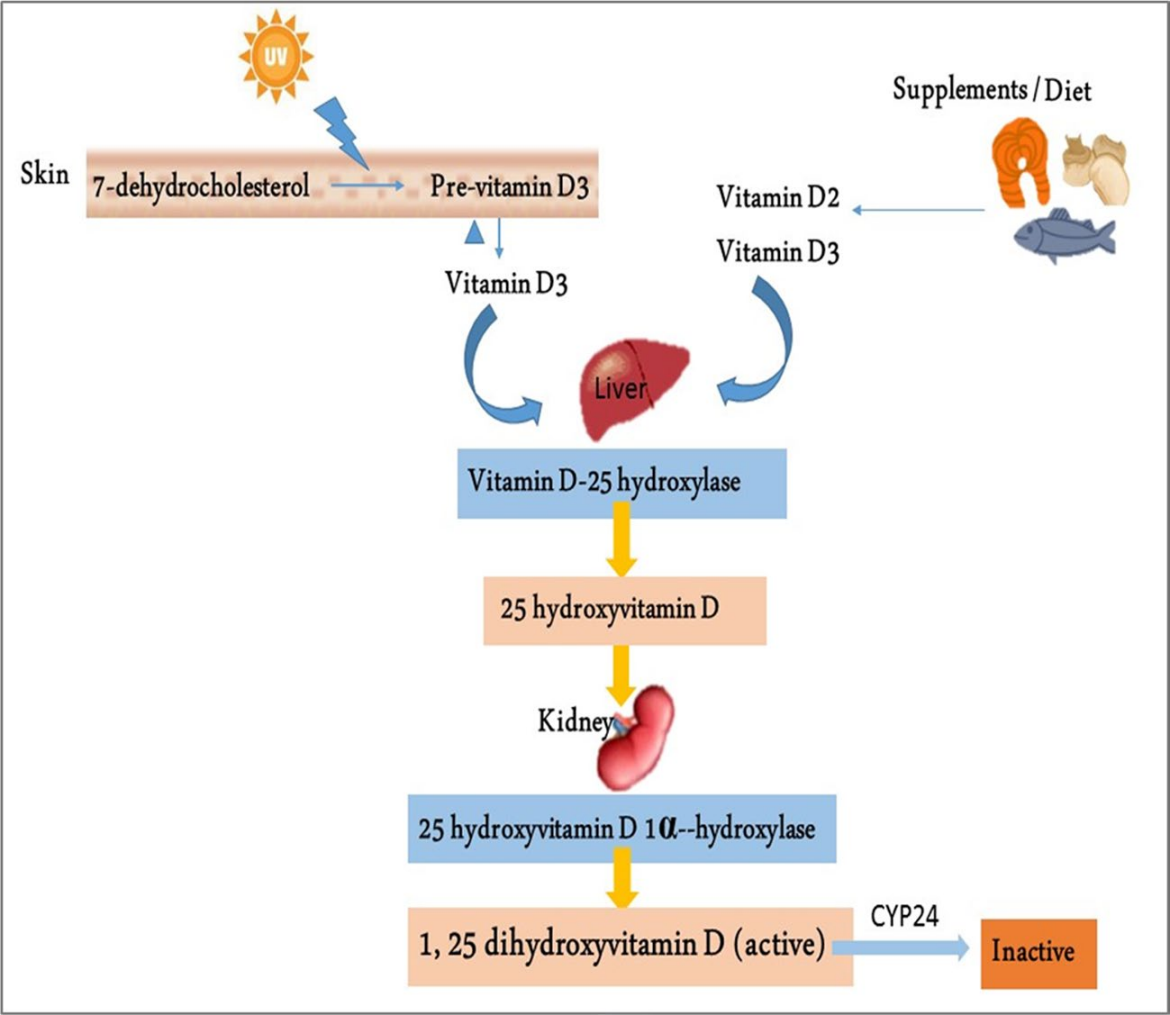
Biosynthesis of vitamin D

Vitamin D (VD) is a fat-soluble vitamin secosteroid derived from diet and skin synthesis during exposure to sunlight (290–315 nm) (Antonucci et al. 2018). VD is biologically inert and must be biologically activated by the hydroxylation process in the liver and kidney (Bikle 2009; Antonucci et al. 2018). VD is converted by 25-hydroxylase in the liver to 25-hydroxyvitamin D (25(OH)D), which is the main circulating form of VD. In the kidney, 25(OH)D is converted by 1-α hydroxylase (CYP2B1) to 1,25-hydroxyvitamin D (1,25(OH)D3), which is the active form of VD (Wacker and Holick 2013). Higher 1,25(OH)D3 concentration is metabolized by 24-hydroxylase to calcitroic acid (Fig. 1). VD exerts its biological action by activating the VD receptor (VDR), a nuclear transcription factor receptor. VDR is a member of the nuclear receptor superfamily. In mammals, VDR is highly expressed in metabolic tissues, such as the intestine, kidney, skin, and thyroid gland, and moderately expressed in nearly all tissues (Norman 2008). Furthermore, VDR is expressed in many malignant tissues. Active VDR binds to vitamin D response elements located in promoter regions of target genes, thereby controlling the transcription of these genes. VDR affects the transcription of at least 913 genes in human SCC25 cells (head and neck squamous cell carcinoma cell line) (Wang et al. 2005). The impacted biological processes range from calcium metabolism to the expression of key antimicrobial peptides. Therefore, it is unsurprising that vitamin D_3_/VDR signaling is involved in mineral and bone homeostasis, modulation of growth, cardiovascular processes, cancer prevention, and regulation of immune responses, including autophagy (Silvagno et al. 2010). Dysfunction of VDR and vitamin D_3_ deficiency can cause poor bone development and health, as well as increase the risk of many chronic diseases, including type 1 diabetes, rheumatoid arthritis, Crohn’s disease, infectious diseases, and cancer (Holick 2010). Besides, VD acts on membrane-associated, rapid response, steroid-binding receptors (MARRS). An alternative membrane receptor for 1,25D_3_ was identified based on binding assays and biochemical purification. Cloning of this receptor provided clues as to the role this new protein might play in 1,25D_3_ signaling. This protein, named 1,25D_3_-MARRS (membrane-associated, rapid response, steroid-binding), is identical to a previously cloned member of the thioredoxin family of proteins, ERp57 (endoplasmic reticulum protein of 57 kDa) or alternatively GRp58 (glucose-regulated protein of 58 kDa)/PDIA3. In addition to localization in the endoplasmic reticulum, 1,25D_3_-MARRS has also been found in the nucleus and contains a domain that can bind to DNA (Wu et al. 2010). One of the best membrane-associated proteins able to bind vitamin D_3_ compounds is the protein disulfide isomerase family A member 3 (Pdia3), also known as MAARS, which has been described as a crucial protein in 1α,25(OH)_2_D_3_-initiated rapid membrane non-genomic signaling pathways (Zmijewski et al. 2020). Activated VDR can also bind the retinoid X receptor (RXR), forming a heterodimer that promotes gene expression through the activation VD response element (Carlberg 2017).

**Fig. 2:**
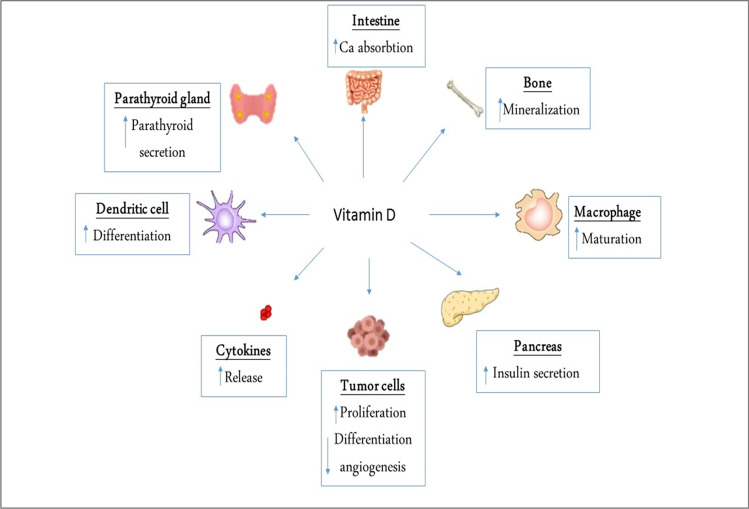
Biological effects of vitamin D

VD regulates calcium and phosphorus homeostasis by regulating the intestinal absorption of calcium and renal tubular phosphate excretion under the parathyroid hormone’s effect (Taylor and Bushinsky 2009). VD is involved in different biological processes in controlling bone mineralization, immune system regulation, and inhibition of tumor progression (Khammissa et al. 2018), as displayed in Fig. 2.

VD controls more than 200 genes that affect various cellular processes. It has an important neuroprotective role in regulating neurotransmission and neuroplasticity (Pirotta et al. 2015). Therefore, VD may affect the pathogenesis and progression of neurodegenerative brain diseases (NBDs), including Parkinson’s disease (PD) (Koduah et al. 2017). There is extensive research concerning the association between VD deficiency and PD. Furthermore, many studies link VD deficiency with the incidence of PD (Zhou et al. 2019). However, there is a controversial point regarding the potential therapeutic benefit of VD supplementation in the management of PD (Barichella et al. 2022). Therefore, this review aims to clarify the preventive and therapeutic roles of VD in PD.

## Parkinson disease

**Fig. 3:**
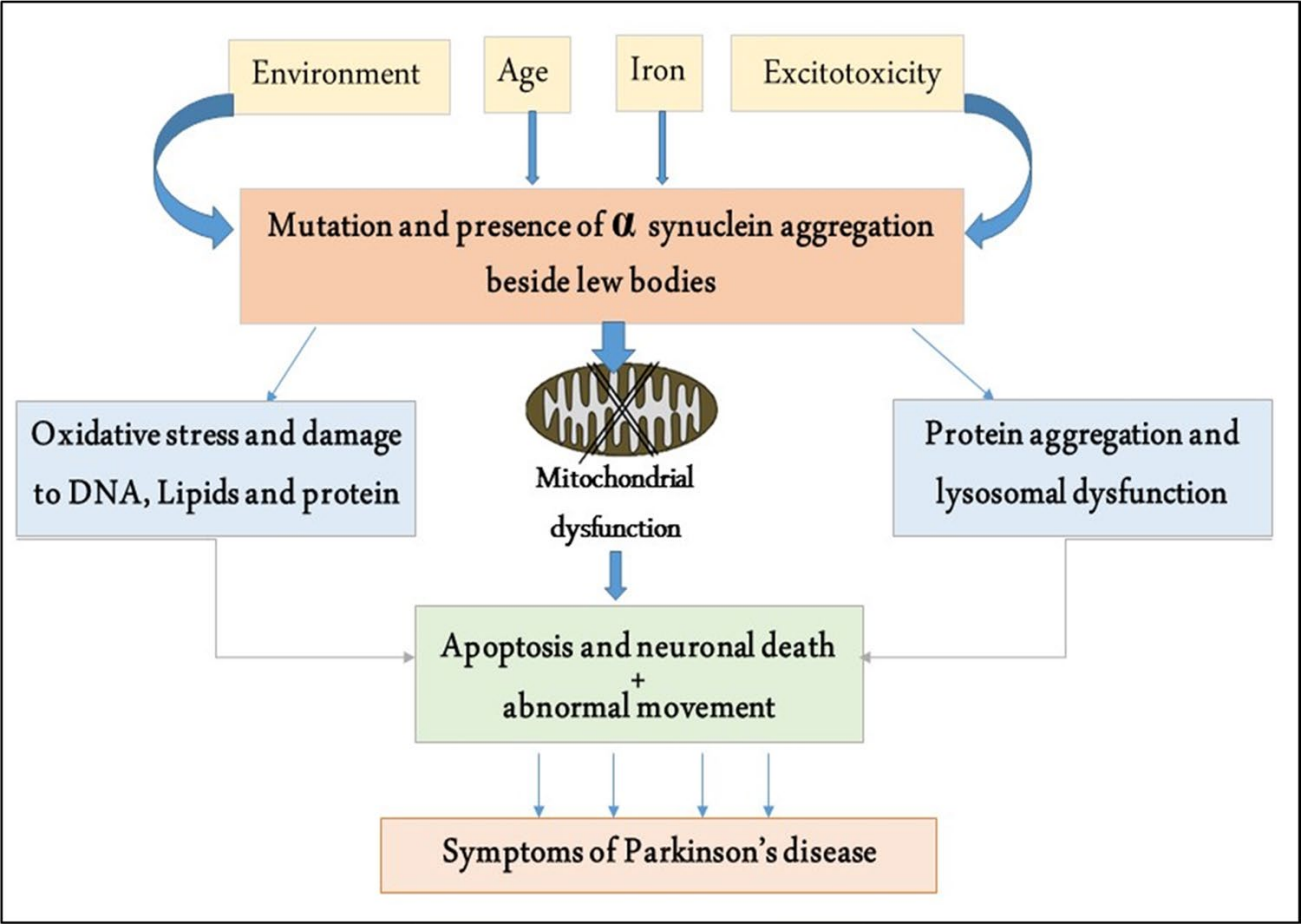
Pathogenesis of Parkinson’s disease

Parkinson’s disease (PD) is the second most common NBD, following Alzheimer’s disease (AD) (Alsubaie et al. 2022). PD was initially identified in 1817 by Doctor James Parkinson, who described shaking palsy. PD progresses due to dopaminergic neuron loss in the substantia nigra (SN) following a great dopamine deficiency in the caudate nucleus and putamen (Alrouji et al. 2023). These changes lead to motor dysfunctions, including rigidity, resting tremors, bradykinesia, and walking difficulty (Poewe et al. 2017). In addition, numerous non-motor disorders are present, including apathy, depression, anxiety, autonomic disorders, dementia, neuropsychiatric disorders, cognitive dysfunction, and sleep disturbances. The incidence of PD in the general population is 0.3% and reaches 4% above the age of 80 years (Savica et al. 2016). The neuropathological characteristic of PD is the deposition of Lewy bodies from aggregated α-synuclein. The deposition of α-synuclein is not limited to the SN but throughout the entire brain, such as the autonomic nervous system (ANS) (Alrouji et al. 2023). Deposition of α-synuclein is progressive for many years before the development of a symptomatic period (Dickson 2018). Actually, the deposition of α-synuclein starts initially in the ANS, mainly in the dorsal motor nucleus of glossopharyngeal and vagus nerves, and then spreads to the other brain areas. These verdicts proposed that PD neuropathology is not limited to SN degeneration. Markedly, in the prodromal phase, non-motor symptoms, including anosmia, constipation, sleep disorders, and depression, develop before dopaminergic degeneration in the SN. Following the development of motor symptoms due to dopaminergic degeneration in the SN, cognitive dysfunctions are propagated due to the involvement of the temporal cortex (Yamasaki et al. 2019). Also, PD is associated with the progression of various inflammatory events linked with the progression of PD neuropathology (Tunold et al. 2021), as presented in Fig. 3.

## VD and dopaminergic neurons

VD and VDR, as well as VD metabolites, can cross BBB and are highly expressed in the human brain that plays a crucial role in maintaining the functions of the CNS (Harms et al. 2011). VDRs are highly expressed in the SN that regulates dopaminergic neurons’ activity and synaptic plasticity (Mayne and Burne 2019). Lima et al. demonstrated that VD had a protective role against the development of PD in rats through attenuation of oxidative stress and neuroinflammation (Lima et al. 2018). Therefore, VD deficiency is associated with the loss of dopaminergic neurons in the SN and an increased risk for new-onset PD (Newmark and Newmark 2007). It has been shown that VD regulates the expression of the tyrosin hydroxylase gene in the dopaminergic neurons of SN with an enhancement of dopaminergic neurotransmission and transporters. It also suppresses the expression of L-type voltage-sensitive Ca2 + channel (LVSC), which supports the enhancement of neurotransmission (Li et al. 2015). An experimental study showed that VD supplementation improves the activity of dopaminergic neurons in rats (Lima et al. 2018). This finding suggests that VD exerts a protective and therapeutic effect against the development of PD by modulating the expression of the tyrosin hydroxylase gene in the dopaminergic neurons of SN. However, due to its anti-inflammatory and antioxidant activities, VD could effectively reduce PD risk.

VD also acts on the endoplasmic reticulum stress protein 57 (ERp57), which is also highly expressed in all brain regions. ERp57 regulates calcium homeostasis in response to neuronal stress and acts as a chaperon in preventing the aggregation of misfolding proteins and the generation of α-synuclein (Lv et al. 2020; Di Risola et al. 2022). In addition, ERp57 improves the expression of redox-sensitive transcription factors to reduce oxidative stress propagation (Grillo et al. 2006). As well, ERp57 protects neurons from amyloid β (Aβ) toxicity (Di Risola et al. 2022), suggesting a protective role of VD/ERp57 in preventing the development of NBDs, including PD. However, the over-expression of ERp57 is not associated with protecting dopaminergic neurons in the SN (Bargsted et al. 2016).

Additionally, VD modulates immunity by decreasing macrophage colony-stimulating factor (M-CSF) and tumor necrosis factor α (TNF-α). Moreover, VD increases the expression of glial cell–derived neurotrophic factor (GDNF), which protects dopaminergic neurons in the SN through its anti-inflammatory and antioxidant effects (Weissmiller and Wu 2012). VD deficiency results in a reduction in the expression of GDNF with further impairment activity of dopaminergic neurons (Zhou et al. 2019; Barichella et al. 2022). Besides, VD exerts a neuroprotective effect through the expression of neuroprotective mediators, including gamma-glutamyl transferase, nuclear erythroid factor 2, and antioxidant genes. VD also prevents the development of neuronal lipid peroxidation and the release of pro-inflammatory cytokines and inducible nitric oxide synthase (iNOS) (Câmara and Brandão 2019). Of note, VD maintains normal calcium homeostasis in the dopaminergic neurons of SN (Berridge 2015). Increased intracellular calcium promotes aggregation of α-synuclein with induction of oxidative stress (Santner and Uversky 2010). Thus, regulation of intracellular calcium by VD inhibits the degeneration of dopaminergic neurons of SN. VD also regulates neuronal concentrations of iron manganese and zinc, thereby preventing metal-induced oxidative stress and neuronal cell death.

Worth mentioning the ability of VD to empower brain growth and development through reducing brain expression of neurotrophin 4 (NT4) and increasing expression of ciliary neurotrophic factor (CNTF), glial cell–derived neurotrophic factor (GDNF), nerve growth factor (NGF), p75 neurotrophin receptors (p75 NTR), transforming growth factor (TGF)-b2, brain-derived neurotrophic factor (BDNF), and neurotrophin 3 (NT3). Thus, VD could halt brain aging (Shirazi et al. 2015).

**Fig. 4:**
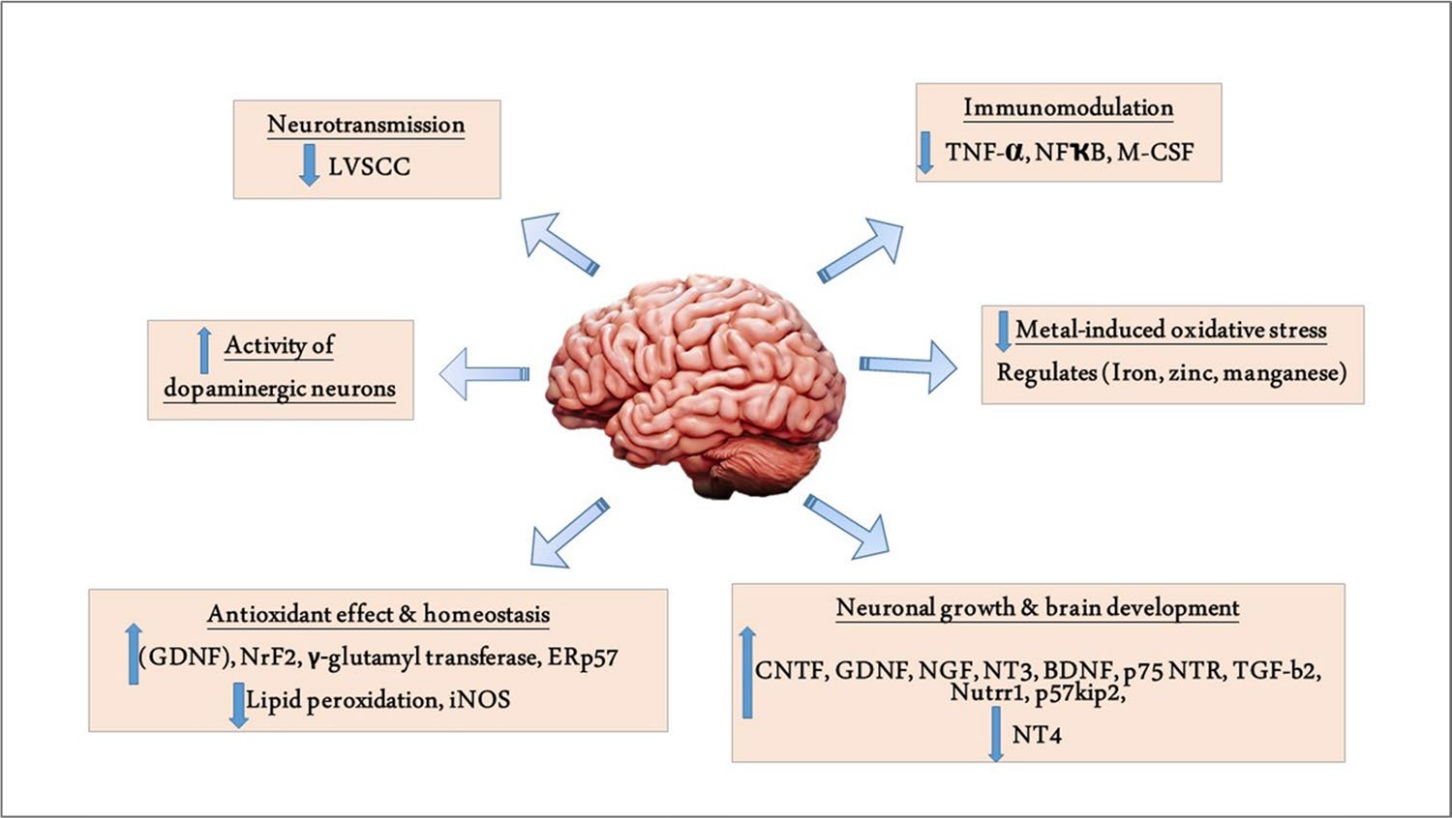
Role of vitamin D in PD. Vitamin D (VD) improves neurotransmission by reducing the L-type voltage-sensitive Ca2^+^ channel (LVSCC). VD increases the dopaminergic neurons’ activity. VD decreases metal-induced oxidative stress. VD has immunomodulatory effects by reducing the expression of tumor necrosis factor α (TNF-α), nuclear factor kappa-light-chain-enhancer of activated B cells (NF-kB), and macrophage colony-stimulating factor (M-CSF). VD improves neuronal homeostasis and has an antioxidant effect by reducing inducible nitric oxide synthase (iNOS) and increasing expression of nuclear erythroid–related factor 2 (NrF2), endoplasmic reticulum stress protein 57 (ERSP57), and γ-glutamyl transferase. VD enhances brain development and neuronal growth by reducing the expression of neurotrophin 4 (NT4) and increasing expression of ciliary neurotrophic factor (CNTF), glial cell–derived neurotrophic factor (GDNF), nerve growth factor (NGF), p75 neurotrophin receptors (p75 NTR), transforming growth factor (TGF)-b2, brain-derived neurotrophic factor (BDNF), and neurotrophin 3 (NT3)

These observations illustrated VD's mechanistic role in the protection and restoration of dopaminergic neurons in the SN (Fig. 4).

## Role of VD in PD

PD neuropathology is affected by VD through genomic and non-genomic pathways, so VD can affect the expression of different genes in the dopaminergic pathway, by which VD is intricate with molecular signaling involved in the progression of PD (Peterson 2014). Different studies revealed VD’s protective effect in managing PD (Muir and Montero‐Odasso 2011; Hiller et al. 2018). Despite symptomatic improvement, PD is a progressive disease not recovered by conventional therapy. However, numerous studies revealed that VD supplementation improves postural stability and gait balance in PD patients (Muir and Montero‐Odasso 2011; Hiller et al. 2018). This beneficial effect might be due to the strengthening of skeletal muscles and spinal cord conductivity (Muir and Montero‐Odasso 2011). A randomized clinical trial showed that VD supplementation might reduce the progression of PD neuropathy for a short period (Suzuki et al. 2013). However, the potential therapeutic effect of VD in well-established PD remains controversial. Thus, there is a debate and conflict about whether treatment with VD can decrease or attenuate the progression of PD or lead to irrespective alleviation in muscle strength.

Furthermore, various studies confirmed a negative association between VD serum levels and PD severity (Suzuki et al. 2012). However, a randomized controlled clinical trial conducted by Suzuki et al. (2013) illustrated that VD supplementation might stabilize PD for a short duration, though this effect of VD was not specific to PD. However, VD serum level is also linked with non-motor symptoms in PD. For example, low VD serum level is associated with cognitive dysfunction and the development of dementia (Feart et al. 2017). A large-scale prospective study of the older French population proposed that preserving normal VD serum levels may prevent cognitive decline and the development of dementia (Feart et al. 2017). Supporting these findings, Peterson et al. (2013) revealed that high VD serum level was correlated with superior cognitive level and mood in PD patients through attenuation of deposition of Aβ. The VD-mediated cognitive enhancing effect is related to the preservation of synaptic plasticity and modulation of neurotransmitter release, as documented by different preclinical studies (Latimer et al. 2014; Phillipson 2017).

Furthermore, cognitive dysfunction is related to the development of olfactory dysfunction in PD patients. VD deficiency is associated with the severity of olfactory dysfunction, which precedes the progression of symptomatic PD (Takeda et al. 2014). Both olfactory and cognitive brain regions are closely related, and olfactory dysfunction like hyposmia predicts the development of PD dementia and motor severity (Yoo et al. 2019; Lee et al. 2021).

Zhou et al. disclosed that VD deficiency might increase the risk for the development of PD (Zhou et al. 2019). However, VD supplementation was not associated with significantly ameliorating motor symptoms in PD patients. A meta-analysis involving eight studies showed that VD serum level  < 20 ng/mL was associated with increased PD risk. VD supplementation did not affect the reduction of PD motor symptoms (Zhou et al. 2019). However, a previous study confirmed that VD supplementation improves PD symptoms (Suzuki et al. 2013). Preclinical studies revealed that VD supplementation reduced PD severity by decreasing the degeneration of dopaminergic neurons in the SN and associated neuroinflammation (Calvello et al. 2017).

In contrast, Shrestha et al. found no association between VD status and PD risk. In a prospective study involving 12,762 participants in relation to VD serum level followed for 17 years, 67 PD cases were identified, and there was no relation to VD status (Shrestha et al. 2016), suggesting that VD deficiency is not a risk factor for the development of PD. A prospective study that included 3173 subjects followed for 29 years revealed that VD supplementation could be protective against the development of PD via modulation of dopaminergic neuron activity (Knekt et al. 2010). However, VD supplementation in established PD might not effectively alleviate PD severity and progression (Chitsaz et al. 2013). VD serum level also does not change during the progression of PD neuropathology (Evatt et al. 2011). Indeed, exposure to sunlight for more than 15 min per week could be a preventive measure against the development of PD (Zhou et al. 2019). Therefore, VD supplementation and sunlight exposure might effectively prevent the development of PD in the high-risk group.

VD acts on nuclear VDR and the MARRS, also known as Erp57/Grp58. ERp57 regulates the activity of dopaminergic neurons in the SN. ERp57 transgenic mice with the neurotoxin 6-OHDA trigger dopaminergic neuron degeneration (Bargsted et al. 2016). ERp57 is upregulated when the UPR is engaged in most experimental systems, suggesting that this foldase may reduce the load of abnormal proteins by enhancing the folding capacity of the ER (Bargsted et al. 2016). It has been demonstrated that altered gene expression of VDR and 1,25D3-MARRS receptors influences vitamin D’s role within neurons and makes them more prone to degeneration (Janjusevic et al. 2022). ERp57 has a synergistic effect and regulates the gene expression mediated by redox-sensitive transcription factors and the adaptive responses of cells to oxidative damage. These findings suggest a neuroprotective effect of VD against PD mediated by 1,25D3-MARRS.

VD insufficiency or deficiency is associated with increased PD risk, but VD supplementation in well-established PD plays an inconsequential role. In this state, preclinical and large-scale prospective studies are recommended.

## Conclusions

PD is a progressive NBD due to dopaminergic neuron loss in the SN following a great dopamine deficiency in the caudate nucleus and putamen. VD and VDR, as well as VD metabolites, are highly expressed in the human brain that plays a crucial role in maintaining the functions of the CNS. VD can cross BBB, and regulates dopaminergic neurons’ activity and synaptic plasticity. VD exerts protective and therapeutic effects against the development of PD by modulating the expression of the tyrosin hydroxylase gene in the dopaminergic neurons of SN. However, due to its anti-inflammatory and antioxidant activities, VD could effectively reduce PD neuropathology. Different studies revealed the protective effect of VD in managing PD. However, the potential therapeutic effect of VD in well-established PD remains controversial. Thus, there is a debate and conflict about whether or not treatment with VD can ameliorate PD. Together, VD insufficiency or deficiency is associated with increased risk. Still, VD supplementation in well-established PD plays a little role. In this state, preclinical and large-scale prospective studies are recommended.

## Data Availability

All data are available in the manuscript.

## References

[CR1] Alrouji M, Al-Kuraishy HM, Al-Gareeb AI, Ashour NA, Jabir MS, Negm WA, Batiha GES (2023) Metformin role in Parkinson’s disease: a double-sword effect. Mol Cell Biochem 1–17 10.1007/s11010-023-04771-7 37266747

[CR2] Alsubaie N, Al-Kuraishy HM, Al-Gareeb AI, Alharbi B, De Waard M, Sabatier J-M, Saad HM, Batiha GE-S (2022). Statins use in alzheimer disease: bane or boon from frantic search and narrative review. Brain Sci.

[CR3] Antonucci R, Locci C, Clemente MG, Chicconi E, Antonucci L (2018). Vitamin D deficiency in childhood: old lessons and current challenges. J Pediatr Endocrinol Metab.

[CR4] Bargsted L, Hetz C, Matus S (2016). ERp57 in neurodegeneration and regeneration. Neural Regen Res.

[CR5] Barichella M, Garrì F, Caronni S, Bolliri C, Zocchi L, Macchione MC, Ferri V, Calandrella D, Pezzoli G (2022). Vitamin D status and Parkinson’s disease. Brain Sci.

[CR6] Berridge MJ (2015). Vitamin D cell signalling in health and disease. Biochem Biophys Res Commun.

[CR7] Bikle D (2009). Nonclassic actions of vitamin D. J Clin Endocrinol Metab.

[CR8] Calvello R, Cianciulli A, Nicolardi G, De Nuccio F, Giannotti L, Salvatore R, Porro C, Trotta T, Panaro MA, Lofrumento DD (2017). Vitamin D treatment attenuates neuroinflammation and dopaminergic neurodegeneration in an animal model of Parkinson’s disease, shifting M1 to M2 microglia responses. J Neuroimmune Pharmacol.

[CR9] Câmara AB, Brandão IA (2019) The relationship between vitamin D deficiency and oxidative stress can be independent of age and gender. Int J Vitam Nutr Res 10.1024/0300-9831/a000614 31711376

[CR10] Carlberg C (2017). Molecular endocrinology of vitamin D on the epigenome level. Mol Cell Endocrinol.

[CR11] Chitsaz A, Maracy M, Basiri K, Izadi Boroujeni M, Tanhaei AP, Rahimi M, Meamar R (2013) 25-hydroxyvitamin d and severity of Parkinson’s disease. Int J Endocrinol 2013 10.1155/2013/689149 PMC3730208 23956745

[CR12] Di Risola D, Ricci D, Marrocco I, Giamogante F, Grieco M, Francioso A, Vasco-Vidal A, Mancini P, Colotti G, Mosca L (2022). ERp57 chaperon protein protects neuronal cells from Aβ-induced toxicity. J Neurochem.

[CR13] Dickson DW (2018). Neuropathology of Parkinson disease. Parkinsonism Relat Disord.

[CR14] Evatt ML, DeLong MR, Kumari M, Auinger P, McDermott MP, Tangpricha V, Investigators PSGD (2011). High prevalence of hypovitaminosis D status in patients with early Parkinson disease. Arch Neurol.

[CR15] Feart C, Helmer C, Merle B, Herrmann FR, Annweiler C, Dartigues J-F, Delcourt C, Samieri C (2017). Associations of lower vitamin D concentrations with cognitive decline and long-term risk of dementia and Alzheimer’s disease in older adults. Alzheimers Dement.

[CR16] Grillo C, D'Ambrosio C, Scaloni A, Maceroni M, Merluzzi S, Turano C, Altieri F (2006). Cooperative activity of Ref-1/APE and ERp57 in reductive activation of transcription factors. Free Radical Biol Med.

[CR17] Harms LR, Burne TH, Eyles DW, McGrath JJ (2011). Vitamin D and the brain. Best Pract Res Clin Endocrinol Metab.

[CR18] Hiller AL, Murchison CF, Lobb BM, O’Connor S, O’Connor M, Quinn JF (2018). A randomized, controlled pilot study of the effects of vitamin D supplementation on balance in Parkinson’s disease: Does age matter?. PLoS ONE.

[CR19] Holick MF (2010) Vitamin D and health: evolution, biologic functions, and recommended dietary intakes for vitamin D. Vitamin D: Physiology, Molecular Biology, and Clinical Applications 3–33

[CR20] Janjusevic M, Gagno G, Fluca AL, Padoan L, Beltrami AP, Sinagra G, Moretti R, Aleksova A (2022). The peculiar role of vitamin D in the pathophysiology of cardiovascular and neurodegenerative diseases. Life Sci.

[CR21] Khammissa R, Fourie J, Motswaledi M, Ballyram R, Lemmer J, Feller L (2018) The biological activities of vitamin D and its receptor in relation to calcium and bone homeostasis, cancer, immune and cardiovascular systems, skin biology, and oral health. BioMed Res Int 2018 10.1155/2018/9276380 PMC5987305 29951549

[CR22] Knekt P, Kilkkinen A, Rissanen H, Marniemi J, Sääksjärvi K, Heliövaara M (2010). Serum vitamin D and the risk of Parkinson disease. Arch Neurol.

[CR23] Koduah P, Paul F, Dörr J-M (2017). Vitamin D in the prevention, prediction and treatment of neurodegenerative and neuroinflammatory diseases. Epma J.

[CR24] Latimer CS, Brewer LD, Searcy JL, Chen K-C, Popović J, Kraner SD, Thibault O, Blalock EM, Landfield PW, Porter NM (2014). Vitamin D prevents cognitive decline and enhances hippocampal synaptic function in aging rats. Proc Natl Acad Sci.

[CR25] Lee JJ, Hong JY, Baik JS (2021). Hyposmia may predict development of freezing of gait in Parkinson’s disease. J Neural Transm.

[CR26] Li H, Jang W, Kim HJ, Jo KD, Lee MK, Song SH, Yang HO (2015). Biochemical protective effect of 1, 25-dihydroxyvitamin D3 through autophagy induction in the MPTP mouse model of Parkinson’s disease. NeuroReport.

[CR27] Lima LA, Lopes MJP, Costa RO, Lima FAV, Neves KRT, Calou IB, Andrade GM, Viana GS (2018). Vitamin D protects dopaminergic neurons against neuroinflammation and oxidative stress in hemiparkinsonian rats. J Neuroinflammation.

[CR28] Lv L, Tan X, Peng X, Bai R, Xiao Q, Zou T, Tan J, Zhang H, Wang C (2020). The relationships of vitamin D, vitamin D receptor gene polymorphisms, and vitamin D supplementation with Parkinson’s disease. Transl Neurodegener.

[CR29] Mayne PE, Burne TH (2019). Vitamin D in synaptic plasticity, cognitive function, and neuropsychiatric illness. Trends Neurosci.

[CR30] Muir SW, Montero-Odasso M (2011). Effect of vitamin D supplementation on muscle strength, gait and balance in older adults: a systematic review and meta-analysis. J Am Geriatr Soc.

[CR31] Newmark HL, Newmark J (2007). Vitamin D and Parkinson’s disease—a hypothesis. Mov Disord.

[CR32] Norman AW (2008). From vitamin D to hormone D: fundamentals of the vitamin D endocrine system essential for good health. Am J Clin Nutr.

[CR33] Peterson AL (2014). A review of vitamin D and Parkinson’s disease. Maturitas.

[CR34] Peterson AL, Murchison C, Zabetian C, Leverenz JB, Watson G, Montine T, Carney N, Bowman GL, Edwards K, Quinn JF (2013). Memory, mood, and vitamin D in persons with Parkinson’s disease. J Parkinsons Dis.

[CR35] Phillipson OT (2017). Alpha-synuclein, epigenetics, mitochondria, metabolism, calcium traffic, & circadian dysfunction in Parkinson’s disease. An integrated strategy for management. Ageing Res Rev.

[CR36] Pirotta S, Kidgell DJ, Daly RM (2015). Effects of vitamin D supplementation on neuroplasticity in older adults: a double-blinded, placebo-controlled randomised trial. Osteoporos Int.

[CR37] Poewe et al. 2017 Poewe W, Seppi K, Tanner C, Halliday G, Brundin P, Volkmann J, Schrag A, Lang A (2017) Parkinson disease. Nat Rev Dis Primers 3:17013 10.1038/nrdp.2017.13 28332488

[CR38] Santner A, Uversky VN (2010). Metalloproteomics and metal toxicology of α-synuclein. Metallomics.

[CR39] Savica R, Grossardt BR, Bower JH, Ahlskog JE, Rocca WA (2016). Time trends in the incidence of Parkinson disease. JAMA Neurol.

[CR40] Shirazi HA, Rasouli J, Ciric B, Rostami A, Zhang G-X (2015). 1, 25-Dihydroxyvitamin D3 enhances neural stem cell proliferation and oligodendrocyte differentiation. Exp Mol Pathol.

[CR41] Shrestha S, Lutsey PL, Alonso A, Huang X, Mosley TH, Chen H (2016). S erum 25-hydroxyvitamin D concentrations in mid-adulthood and Parkinson’s disease risk. Mov Disord.

[CR42] Silvagno F, Poma CB, Realmuto C, Ravarino N, Ramella A, Santoro N, D'Amelio P, Fuso L, Pescarmona G, Zola P (2010). Analysis of vitamin D receptor expression and clinical correlations in patients with ovarian cancer. Gynecol Oncol.

[CR43] Suzuki M, Yoshioka M, Hashimoto M, Murakami M, Kawasaki K, Noya M, Takahashi D, Urashima M (2012). 25-hydroxyvitamin D, vitamin D receptor gene polymorphisms, and severity of Parkinson’s disease. Mov Disord.

[CR44] Suzuki M, Yoshioka M, Hashimoto M, Murakami M, Noya M, Takahashi D, Urashima M (2013). Randomized, double-blind, placebo-controlled trial of vitamin D supplementation in Parkinson disease. Am Clin Nutr.

[CR45] Takeda A, Baba T, Kikuchi A, Hasegawa T, Sugeno N, Konno M, Miura E, Mori E (2014). Olfactory dysfunction and dementia in Parkinson’s disease. J Parkinsons Dis.

[CR46] Taylor JG, Bushinsky DA (2009). Calcium and phosphorus homeostasis. Blood Purif.

[CR47] Tunold J-A, Geut H, Rozemuller JA, Henriksen SP, Toft M, Van de Berg WD, Pihlstrøm L (2021). APOE and MAPT are associated with dementia in neuropathologically confirmed Parkinson’s disease. Front Neurol.

[CR48] Wacker M, Holick MF (2013). Sunlight and Vitamin D: a global perspective for health. Dermato-endocrinology.

[CR49] Wang T-T, Tavera-Mendoza LE, Laperriere D, Libby E, Burton MacLeod N, Nagai Y, Bourdeau V, Konstorum A, Lallemant B, Zhang R (2005). Large-scale in silico and microarray-based identification of direct 1, 25-dihydroxyvitamin D3 target genes. Mol Endocrinol.

[CR50] Weissmiller AM, Wu C (2012). Current advances in using neurotrophic factors to treat neurodegenerative disorders. Transl Neurodegener.

[CR51] Wu W, Beilhartz G, Roy Y, Richard CL, Curtin M, Brown L, Cadieux D, Coppolino M, Farach-Carson MC, Nemere I (2010). Nuclear translocation of the 1, 25D3-MARRS (membrane associated rapid response to steroids) receptor protein and NFκB in differentiating NB4 leukemia cells. Exp Cell Res.

[CR52] Yamasaki TR, Holmes BB, Furman JL, Dhavale DD, Su BW, Song E-S, Cairns NJ, Kotzbauer PT, Diamond MI (2019). Parkinson’s disease and multiple system atrophy have distinct α-synuclein seed characteristics. J Biol Chem.

[CR53] Yoo HS, Chung SJ, Lee YH, Ye BS, Sohn YH, Lee PH (2019). Olfactory anosognosia is a predictor of cognitive decline and dementia conversion in Parkinson’s disease. J Neurol.

[CR54] Zhou Z, Zhou R, Zhang Z, Li K (2019) The association between vitamin D status, vitamin D supplementation, sunlight exposure, and Parkinson’s disease: a systematic review and meta-analysis. Med Sci Monit Int Med J Exp Clin Res 25:666 10.12659/MSM.912840 PMC6352758 30672512

[CR55] Zmijewski MA, Carsten C (2020) Vitamin D receptor (s): In the nucleus but also at membranes?. Experimental Dermatology 9:876–884 10.1111/exd.14147 32654294

